# Role of MAML1 in targeted therapy against the esophageal cancer stem cells

**DOI:** 10.1186/s12967-019-1876-5

**Published:** 2019-04-16

**Authors:** Meysam Moghbeli, Hooman Mosannen Mozaffari, Bahram Memar, Mohammad Mahdi Forghanifard, Mehran Gholamin, Mohammad Reza Abbaszadegan

**Affiliations:** 1 Department of Modern Sciences and Technologies, Faculty of Medicine, Mashhad University of Medical Sciences, Mashhad, Iran; 2 Gastroenterology and Hepatology Research Center, Mashhad University of Medical Sciences, Mashhad, Iran; 3 Surgical Oncology Research Center, Mashhad University of Medical Sciences, Mashhad, Iran; 4 Department of Biology, Damghan Branch, Islamic Azad University, Damghan, Iran; 5 Department of Laboratory Sciences, School of Paramedical Sciences, Mashhad University of Medical Sciences, Mashhad, Iran; 6 Immunology Research Center, Mashhad University of Medical Sciences, Mashhad, Iran

**Keywords:** Cancer stem cell, CD44, ESCC, NOTCH pathway, MAML1, ABC transporter

## Abstract

**Background:**

Esophageal cancer is the sixth-leading cause of cancer-related deaths worldwide. Cancer stem cells (CSCs) are the main reason for tumor relapse in esophageal squamous cell carcinoma (ESCC). The NOTCH pathway is important in preservation of CSCs, therefore it is possible to target such cells by targeting MAML1 as the main component of the NOTCH transcription machinery.

**Methods:**

In present study we isolated the CD44+ ESCC CSCs and designed a MAML1-targeted therapy to inhibit the NOTCH signaling pathway. CSCs were isolated using magnetic cell sorting utilizing the CD44 cell surface marker. Several stem cell markers were analyzed in the levels of protein and mRNA expression. The isolated CSCs were characterized in vivo in NUDE mice. Biological role of MAML1 was assessed in isolated CD44+ CSCs. A drug resistance assay was also performed to assess the role of MAML1 in CD44+ CSCs with 5FU resistance.

**Results:**

The CD44+ CSCs had ability to form tumors in NUDE mice. MAML1 silencing caused a significant decrease (p = 0.019) and ectopic expression caused a significant increase in migration of CD44+ CSCs (p = 0.012). Moreover, MAML1 silencing and ectopic expression significantly increased and decreased 5FU resistance, respectively (p < 0.05). MAML1 silencing significantly increased the number of cells in G1 phase (p = 0.008), and its ectopic expression significantly increased the number of CD44+ CSCS in S phase (p = 0.037).

**Conclusions:**

MAML1 may be utilized for targeted therapy with a low side effect to eliminate the CD44+ CSCs through inhibition of canonical NOTCH pathway in ESCC patients.

## Background

Esophageal cancer (EC) is the sixth-leading cause of cancer related deaths worldwide [1, 2]. There are two histopathological EC subtypes; esophageal adenocarcinoma (EAC) and esophageal squamous cell carcinoma (ESCC). Patients with either subtype receive the same treatment, which is a neo adjuvant chemo-radiotherapy (nCRT) before the surgery. A heterogeneous response occurs to nCRT; approximately 75% of EAC patients will not achieve a pathological complete response [3]. Apart from new progresses in therapeutic modalities, ESCC patients have poor prognoses due to chemo and radiation therapy resistance. The 5-year survival rate after surgery is 20–40% [4]. Although, specific multimodal treatments increase the 5-year survival [3], 60–70% of patients do not respond to these treatments [5]. Cancer stem cells have been proposed as important factors in resistance to the multimodal treatments [6, 7]. Cancer stem cells have been shown to have several specific characteristics including drug resistance, self-renewal, and tumorigenicity [8]. They can be identified using intracellular and cell surface markers [8]. CD44 is an integral membrane glycoprotein that binds to hyaluronic acid and is involved in tumor growth and metastasis [9]. CD44 is one of the main cell surface candidates in cancer stem cells detection and isolation [10]. Recent work has shown that CD44 is involved in self-renewal and contributes to reactive oxygen species (ROS) depletion by up regulation of glutathione as an important antioxidant [11]. CD44 has been introduced as one of the main cell surface markers in epithelial CSCs [11, 12]. Some CD44+ CSCs have epithelial–mesenchymal transition (EMT) ability, and contribute to tumor progression and metastasis [13, 14]. NOTCH signaling is involved in self-renewal and cell fate through diverse processes including vascular development, hematopoiesis, and neurogenesis [15]. NOTCH is a critical signaling pathway involved in CSC regulation [16]. This range of functions is related to the ability of NOTCH pathway to regulate cell proliferation, apoptosis, and differentiation [17]. Four NOTCH receptors (Notch1-4) and six ligands (Jagged1-2, Delta1, and Delta-like 1-4) are presented in mammalian cells. Receptor–ligand interactions between adjacent cells trigger the NOTCH pathway via at least two proteolytic cleavages at S2 and S3 sites of NOTCH receptor. These cleavages are mediated by ADAM metalloprotease and gamma secretase activity to release the intracellular domain of NOTCH (ICN) [18]. ICN migrates to the nucleus and binds to CSL family of DNA-binding transcription factors. Subsequently, CSL activation is done by substitution of transcriptional co-repressors, including CIR [19], SMRT/N-CoR [20], and KyoT2 [21], and recruitment of co-activators, including CBP/p300 [22] and mastermind-like proteins (MAML) [23]. The multiprotein complex comprising MAML1, CSL, and ICN activates transcription of NOTCH target genes following the activation of NOTCH receptors [24]. HES and HEY proteins are the main targets of the NOTCH pathway and act as transcriptional repressors through their basic helix-loop-helix and WRPW domains to regulate several genes including cyclin D1 [25], NF-κB [26], p21 [17], MYC [27], and SLUG [28]. Despite the pivotal role of the NOTCH pathway in tissue homeostasis, over-activation of stem cell pathways can result in malignant cells. The NOTCH pathway increases tumor survival by maintaining CSCs and is involved in chemotherapeutic resistance and EMT. Generally, targeting the NOTCH pathway can be effective in limiting tumor recurrence. In this study, we hypothesized that the CSCs may be the main reason for chemo-radio therapeutic resistance in ESCC patients. Cancer stem cells were isolated and characterized from an ESCC patient and the role of MAML1 as the main component of the NOTCH pathway was assessed in the biology of isolated ESCC-CSCs for the first time.

## Methods

### Cell culture and sphere formation

Fresh tissue was obtained from an 84-year old male ESCC patient who was undergone an esophagectomy before chemo-radio therapy. Informed consent form which was approved by the ethic committee was filled by the patient. The sample was minced into 1 mm pieces and transferred to D-Hank’s buffer containing collagenase type I (0.25 mg/ml; Worthington Biomedical Corporation) and incubated at 37 °C for 3 h. Single cells were cultured in a specific complete medium comprising DMEM/F12 supplemented with penicillin/streptomycin (10 units/ml), insulin (25 µg/ml), epidermal growth factor (EGF, 20 ng/ml), and basic fibroblast growth factor (bFGF, 10 ng/ml) in a humidified atmosphere containing 5% CO_2_ at 37 °C. For tumor sphere formation, cancer cells were cultured for 3 weeks in low attachment plates.

### RNA isolation and real-time PCR

Total RNA was extracted using Trizol according to the manufacturer’s instructions. cDNA synthesis was performed using the First-Strand Synthesis kit (Fermentas, Lithuania). Subsequently, comparative relative real-time PCR was performed in triplicate reactions using SYBR Green (GENETBIO, Korea) by a Stratagene Mx3000P real-time thermocycler (Stratagene, La Jolla, CA). Glyceraldehyde-3-phosphate dehydrogenase (GAPDH) was used as a normalizer (Table 1). Greater than twofold decreases or increases in mRNA expression were considered as under- and over-expression, respectively.

**Table 1:** Primer sequences for the real time PCR

	Sequence	Thermal profile	Size (bp)
HES1	F: CCCAACGCAGTGTCACCTTC R: TACAAAGGCGCAATCCAATATG	95 °C(10 min)[95 °C(30 s)/58 °C(30 s)/72 °C(30 s)]40	304
GAPDH	F: GGAAGGTGAAGGTCGGAGTCA R: GTCATTGATGGCAACAATATCCACT	95 °C(10 min)[95 °C(30 s)/58 °C(30 s)/72 °C(30 s)]40	108
HEY1	F: ACGGCAGGAGGGAAAGGTTAC R: CTGGGAAGCGTAGTTGTTGAGATG	95 °C(10 min)[95 °C(30 s)/58 °C(30 s)/72 °C(30 s)]40	294
HEY2	F: AGAAAAGGAGAGGGATTATAGAGAAAAGG R: AGCGTGTGCGTCAAAGTAGC	95 °C(10 min)[95 °C(30 s)/58 °C(30 s)/72 °C(30 s)]40	300
Nanog	F: GCAATGGTGTGACGCAGAAGGC R: GCTCCAGGTTGAATTGTTCCAGGTC	95 °C(10 min)[95 °C(30 s)/65 °C(30 s)/72 °C(30 s)]40	137
Bmi1	F: CGTGTATTGTTCGTTACCTGGAGAC R: CATTGGCAGCATCAGCAGAAGG	95 °C(10 min)[95 °C(30 s)/62 °C(30 s)/72 °C(30 s)]40	204
CD44s	F: TCCAACACCTCCCAGTATGACA R: GGCAGGTCTGTGACTGATGTACA	95 °C(10 min)[95 °C(15 s)/60 °C(60 s)]40	83
CD44v3	F: GCACTTCAGGAGGTTACATC R: CTGAGGTGTCTGTCTCTTTC	95 °C(10 min)[95 °C(15 s)/60 °C(60 s)]40	181
CD44v6	F: AGGAACAGTGGTTTGGCAAC R: CGAATGGGAGTCTTCTCTGG	95 °C(10 min)[95 °C(15 s)/60 °C(30 s)]40	68
MAML1	F: TCTCGCGGAACAGGAGA R: GCAGCAGAGGACCCTGTG	95 °C(10 min)[95 °C(30 s)/58 °C(30 s)/72 °C(30 s)]40	123

### In vivo tumorigenicity assay

Six-week-old male nude mice (NUDE) were used in this study. Four million tumor cells derived from the ESCC spheres were mixed with Matrigel (2:1, volume), and mice were injected subcutaneously. Tumor growth was monitored weekly and measured using a caliper.

### Western blot, H&E staining, and immunohistochemistry of xenografts and primary tumors

Proteins were extracted with RIPA buffer, separated on 10% SDS-PAGE, transferred to membranes (Millipore, Bedford, MA), and immunoblotted with specific antibodies. The explanted tumor was embedded in paraffin and cut into 4 mm sections using a microtome. Sections were de-paraffinized using Roticlear (Carl Roth, Germany) and hydrated in an ethanol series. Antigens were retrieved by heating the samples in 10 mM citrate buffer. The samples were blocked and then incubated at 4 °C for 16 h with the primary antibodies: anti-P63 (1:1000, BD Biosciences), anti-cytokeratin 18 (1:500, DakoCytomation), anti-cytokeratin 19 (1:500, Miltenyi Biotech), anti-CD117 (1:2000, Dako), or anti-CD44 (1:1000, Dako), and then with secondary antibody.

### Immunocytochemistry

Cells (10^6^ cells per slide) were plated and cultured for 24 h on sterile slides. The slides were washed three times with phosphate-buffered saline (PBS, 0.01 M, pH 7.4), and fixed in 4% formaldehyde for 10 min at room temperature, and incubated in 3% hydrogen peroxide for 10 min. Slides were incubated with anti-p63 antibody (1:500, Abcam), anti-cytokeratin 18 (1:500, Novocastra), and anti-CD44 (1:1000, Dako) overnight at 4 °C. Finally, the slides were incubated with secondary antibodies for 30 min at 37 °C and visualized with diaminobenzidine.

### Cell cycle analysis

CD44+ CSCs were plated and transfected in 24 wells plates. The transfected cells were trypsinized and mixed with propidium iodide (PI, 50 µg/ml) and RNaseA (100 µg/ml) and incubated at 4 °C for 60 min. The cells were washed with PBS to eliminate the extra PI. Finally, a suspension of 300,000 cells/ml was prepared to detect cell cycle distribution using flow cytometry (Franklin Lakes, USA). The cell cycle was analyzed using Mod Fit LT (version 4.1) software.

### Plasmids and transfection

Human full-length MAML1 cDNA was sub-cloned into the CMV2-pFLAG vector (a generous gift from Dr. Lizi Wu, University of Florida). CD44+ CSCs were transfected with X-treme GENE HP (Roche, Germany) according to the manufacturer’s instructions. Cells were also transfected with pLKO vector, harboring MAML1 short hairpin RNA for 24 h (MAML1 shRNA-1790). ShRNA encoding target sequences to MAML1 mRNA was designed by the primer sequences as follows: 5′-CCGGTCCGGGCTGGACTACGGCAATACAAACTCGAGTTTGTATTGCCGTAGTCCAGCTTTTT-3′ and 5′-AATTCAAAAAGCTGGACTACGGCAATACAAACTCGAGTTTGTATTGCCGTAGTCCAGCCCGG-3′.

### Drug resistance assay

CD44+ CSCs cells were transfected with the MAML1-CMV2-pFLAG and MAML1-pLKO vectors using X-treme GENE transfection reagent. Cells were exposed to varying concentrations of 5FU (10^−2^ µm/l to 10^3^ µm/l), treated with 50 µl of 5 mg/ml of dimethyl thiazolyldiphenyltetrazolium (MTT) dye (Sigma; Dorset) and incubated for 4 h at 37 °C. The plates were quantified at an absorbance of 570 nm. All assays were repeated three times with 10 replicates.

### Migration assay

Cells were cultured in specific serum-free medium in 6-well plates and grown to achieve 70% of confluence. The medium was replaced 24 h after transfection. After 10 days, the cells were scratched with a p200 pipette tip and free detached cells were removed with PBS. Plates were monitored for 15 days in 5 days intervals. The cell-free area was measured using a 10× objective (OPTIKA, Italy). Non transfected cells were used as control to assess the migration of transfected cells. All assays were performed in triplicates and significant changes among transfected and non-transfected cells were assessed by ANOVA test.

## Results

### Characterization of isolated CD44+ CSCs

#### Sphere formation

**Fig. 1:**
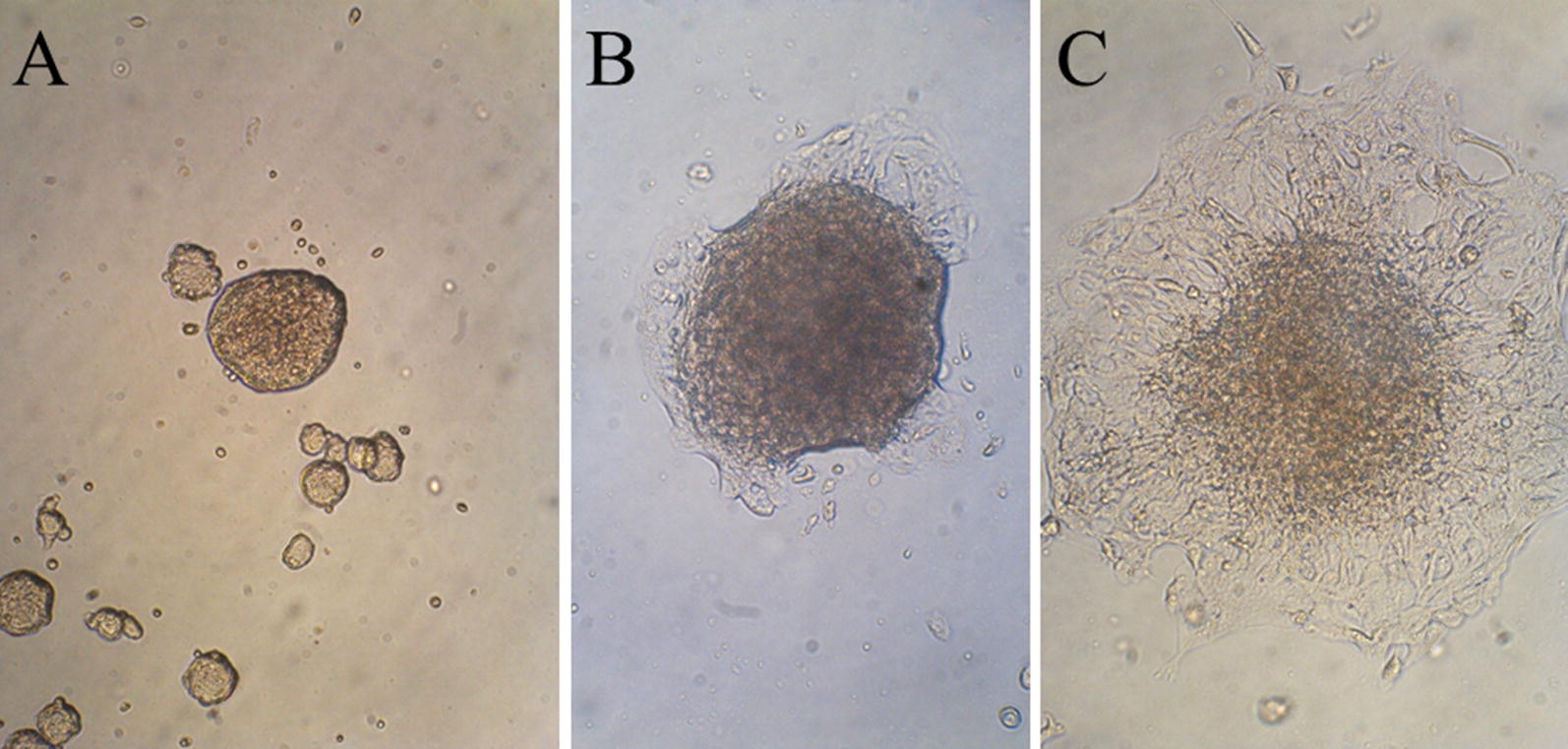
Sphere formation and differentiation. The first CD44+ tumor sphere was observed after 10 days (low adherent flasks) (**A**). Spheres were able to adhere and differentiate in the adherent flasks (**B**, **C**)

The first step in characterization of isolated CSCs was tumor sphere formation in specific medium. After the enzymatic digestion, cells were cultured in specific medium in low adherent flasks, which inhibit fibroblast overgrowth. The first CD44+ tumor sphere was observed after 10 days (Fig. 1A). Spheres were able to adhere and differentiate in the flasks (Fig. 1B, C).

#### CD44+ CSC enrichment and NUDE mice injection

**Fig. 2:**
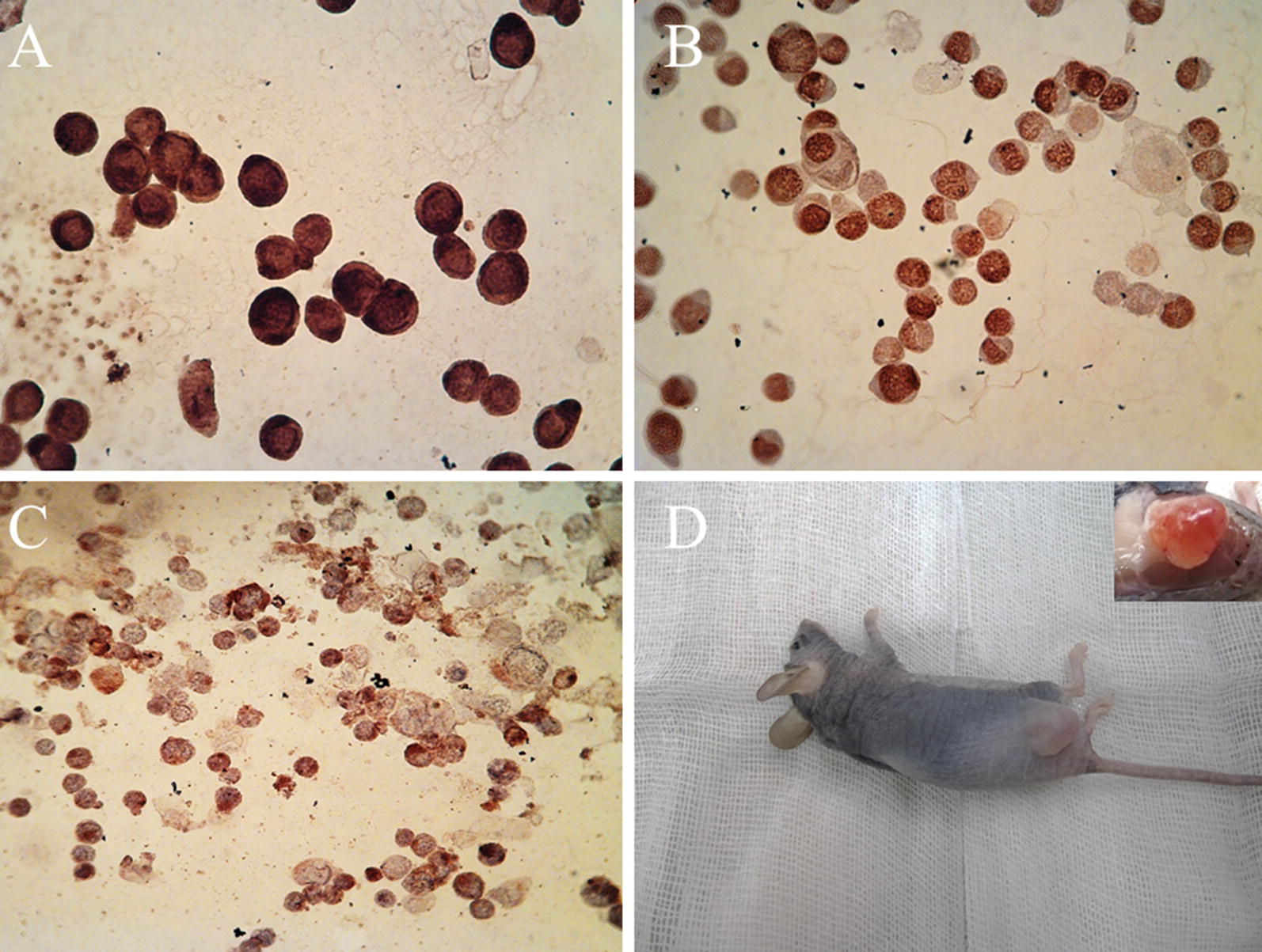
Immunocytochemistry was performed before the mice injection to ensure about the characteristics of isolated cells. The cells were positive for the CK18 as one of the main epithelial markers (**A**). The cells were also positive in the case of P63 expression as the well know esophageal marker (**B**). Finally, it was shown that the cells had CD44 expression, approving the isolation process based on CD44 (**C**). In vivo assay was done using the CD44+ CSCs injection to the nude mice. Mice were sacrificed and tumor was resected after 45 days (1.4 cm) (**D**)

A major factor in characterization of CSCs is tumor generation in NUDE mice. Cancer stem cell biomarkers were characterized using immunocytochemistry for P63, CD44, and CK18. The CSCs expressed all these markers, indicating they were of esophageal origin (Fig. 2A–C). The CD44+ CSCs were cultured in low-adherent plates and four million cells were injected into the mice using Matrigel. We first observed the tumor after 12 days, and allowed it to grow for 45 days. The tumor was 1.4 by 1 mm after the resection (Fig. 2D).

#### Expressional analysis of stem cell markers

**Fig. 3:**
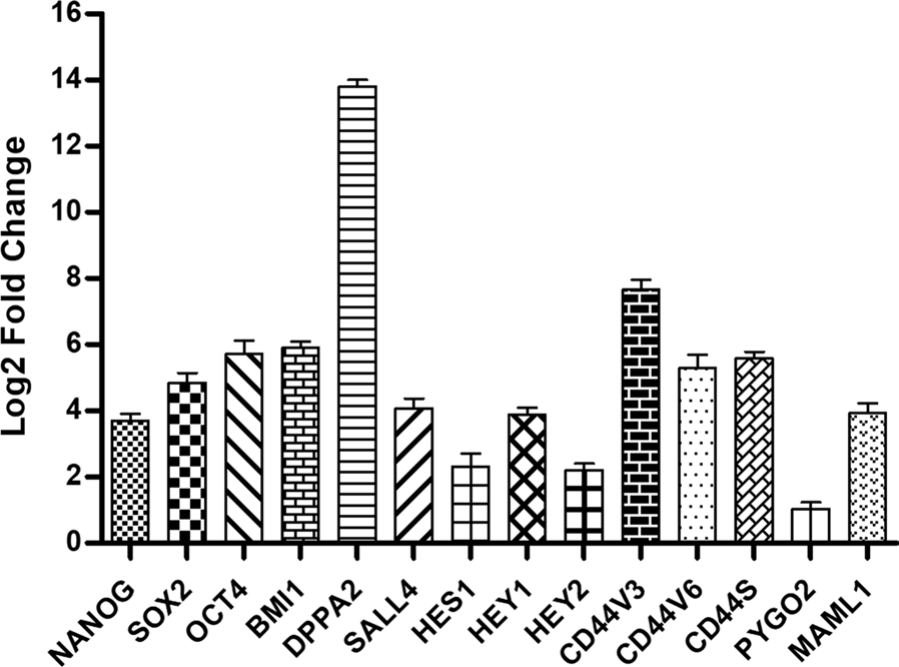
Expressional analysis of stem cell markers. Compared to the control cells (CD44−), the CD44+-enriched CSCs overexpressed NANOG, SOX2, and OCT4. Moreover, the isolated CD44+ cells overexpressed SALL4, DPPA2, and BMI1. Additionally, CD44V3, CD44V6, and CD44S, three CD44 isoforms were overexpressed in these CSCs. MAML1 a major factor in the transcription mechanism, and the NOTCH target genes HES1, HEY1, and HEY2 were overexpressed. Expression of PYGO2, a major component of the WNT transcription mechanism, was normal

To ensure that the CD44+ CSCs have stem cell features, we analyzed mRNA expression and compared it to CD44− cells as the control. Compared to the control cells, the CD44+-enriched CSCs had NANOG, SOX2, and OCT4 over expression. Moreover, the isolated CD44+ cells had SALL4, DPPA2, CD44V3, CD44V6, CD44S, and BMI1 over expression. These results approved the efficiency of our isolation procedure for CD44+ CSCs. Finally, we tested the expression of NOTCH components in the CD44+ enriched cells. MAML1 and NOTCH target genes (HES1, HEY1, and HEY2) were over expressed. Expression of PYGO2 as an important component of the WNT signaling pathway was normal. This showed that the WNT pathway probably has no significant role in the biology of CD44+ enriched cells. DPPA2 and PYGO2 had the highest and lowest levels of mRNA expression with 13.8- and 1.03-fold changes, respectively (Fig. 3).

#### Expressional analysis after the NUDE mice tumor resection

**Fig. 4:**
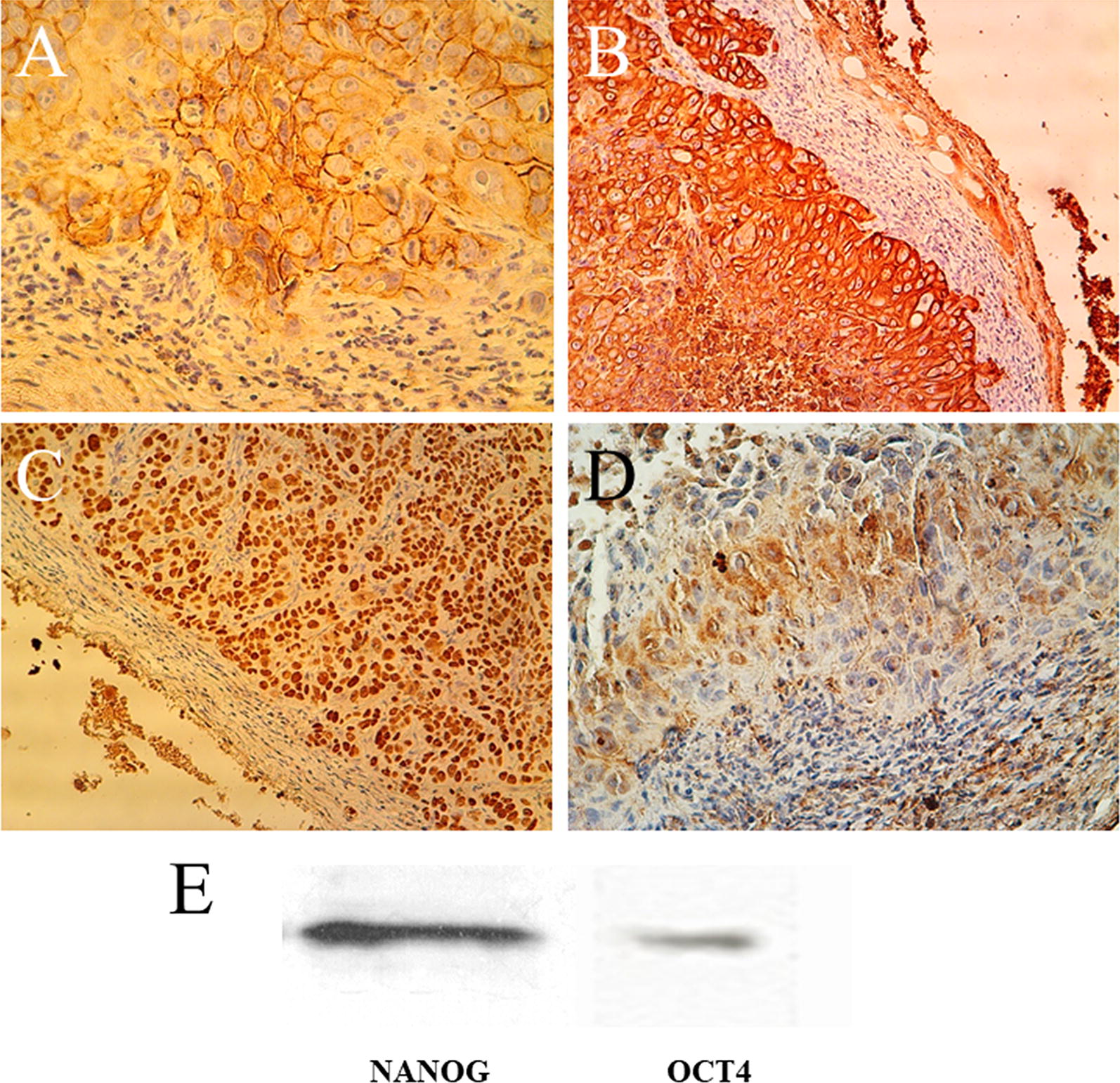
Expressional analysis after the NUDE mice tumor resection. Expression of CD44 in the resected tumor from the nude mice (**A**). Representative IHC analyses of CK18 in ESCC tissues (**B**). Strong expression of P63 (**C**). SOX2 expression (**D**). NANOG and OCT4 protein expression were analyzed using the western blot in ESCC tumor which was resected from the nude mice (**E**)

After the resection, tumor tissue was evaluated to determine the ratio of cancer/normal cells by H&E staining. Protein expression was analyzed for P63, CD44, CK18, CK19, and SOX2 using immunohistochemistry (Fig. 4A–D). Moreover, NANOG and OCT4 expression were analyzed by western blot (Table 2) (Fig. 4E). These results indicated that the generated tumor in nude mice was ESCC with a self-renewal behavior.

**Table 2:** Antibodies for western blot

Antibody	Info
Oct4	Anti-Oct4 antibody (ab200834), rabbit monoclonal
Nanog	Anti-Nanog antibody (ab109250), rabbit monoclonal
β-Actin	Anti-beta actin antibody (ab25894), rabbit polyclonal to beta actin

### Role of MAML1 in biology of CD44+ CSCs

#### Transfection and expressional analysis

**Fig. 5:**
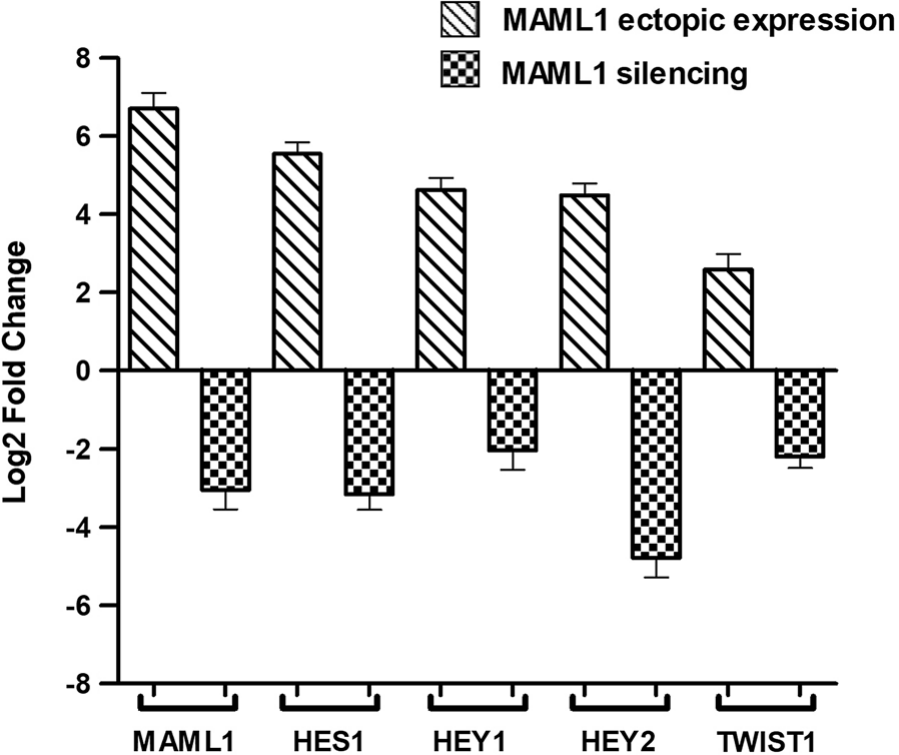
Expression analysis of NOTCH target genes in the level of mRNA expression after the transfection approved the efficiency of transfection. Red shows the fold changes after ectopic expression and blue represents the fold changes following the silencing

In the present study we assessed the probable role of MAML1 in the preservation of CD44+ CSCs. Therefore, MAML1, as the main component of the NOTCH transcription machinery, was targeted by ectopic expression and silencing. MAML1 mRNA expression was evaluated after the ectopic expression and silencing with real time PCR. MAML1 mRNA expression in CD44+ transfected cells was compared to that of the CD44+ non transfected cells. MAML1 expression was 6.71-fold greater and 3.04-fold less in ectopically-expressed and silenced CD44+ CSCs, respectively. Moreover, to assess the expected change in MAML1 function, mRNA expression of HEY1, HEY2, and HES1 as the main targets of the NOTCH pathway were also assessed after the transfection (Fig. 5).

#### Migration assay

**Fig. 6:**
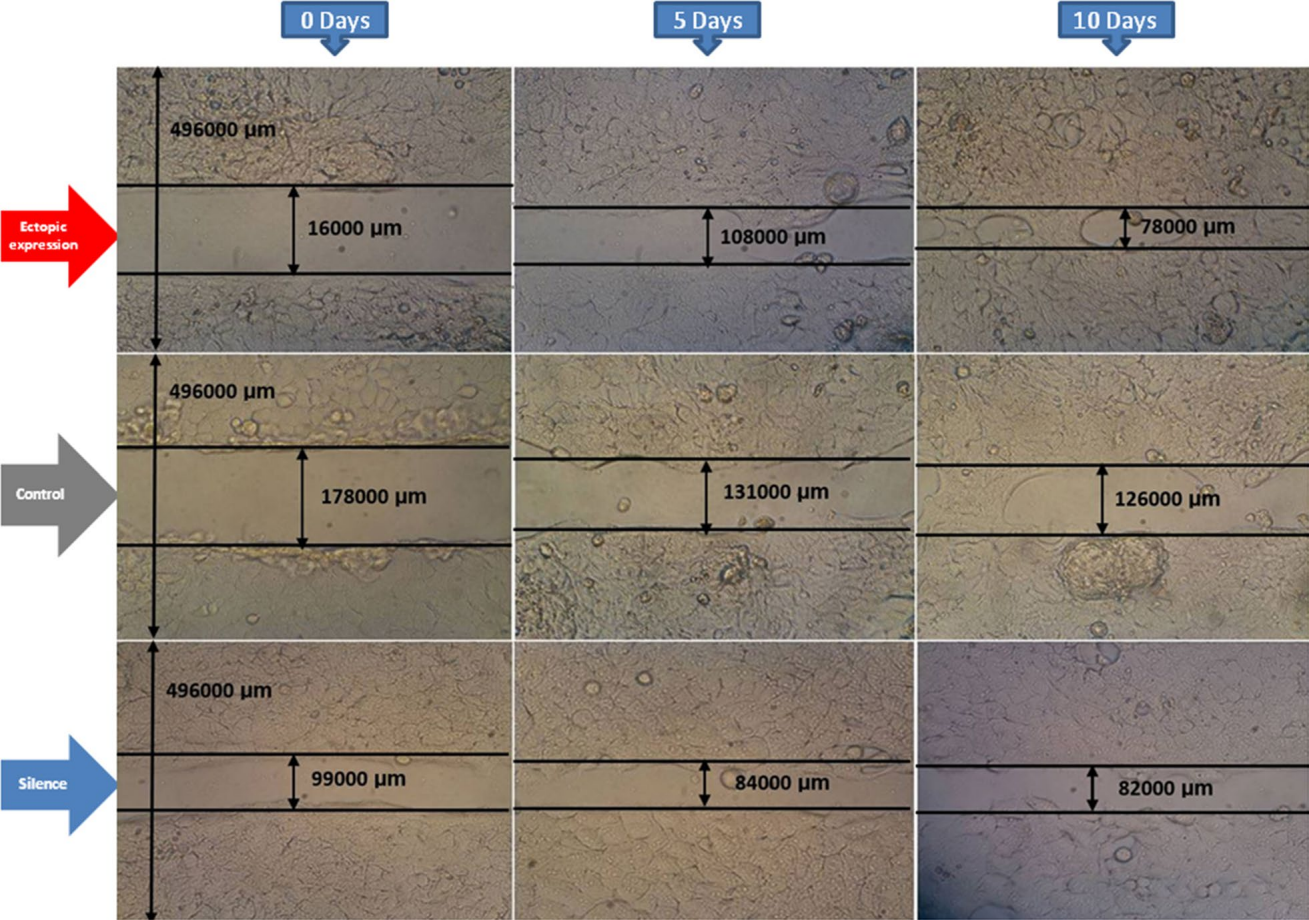
Migration assay was performed in transfected and non-transfected CD44+ CSCs. The cells were monitored in 5 days intervals (×10 objective; OPTIKA, Italy)

**Fig. 7:**
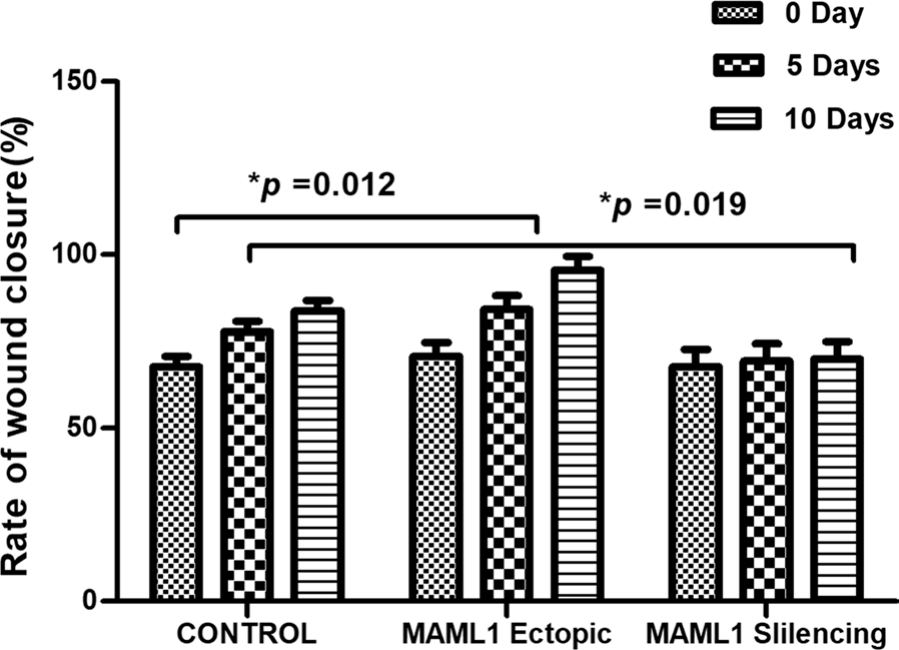
There was a significant increase and decrease in cell migration after the MAML1 ectopic expression and silencing, respectively

One of the most important features of the CSCs is their ability to migrate and cause metastasis via EMT. To assess this, a scratch assay was performed to analyze the probable role of the NOTCH pathway in the regulation of cell migration. The results showed that CSC migration increased significantly after the MAML1 ectopic expression (p = 0.012). Migration of CSCs decreased after the MAML1 silencing (p = 0.019) (Figs. 6, 7). Two involved factors in the EMT process are Twist and Snail. We previously showed that MAML1 and Twist1 expression in ESCC patients is significantly correlated with EMT [29]. Twist1 expression was also assessed after the migration assay in transfected cells. Interestingly, Twist1 expression decreased 2.18-fold and increased 2.59-fold in silenced and ectopically expressed cells, respectively, relative to the non-transfected cells. Therefore, similar to the recent report regarding the role of MAML1/TWIST1 in EMT in ESCC patients, such markers are also involved in EMT of CD44+ CSCs.

#### Drug resistance

**Fig. 8:**
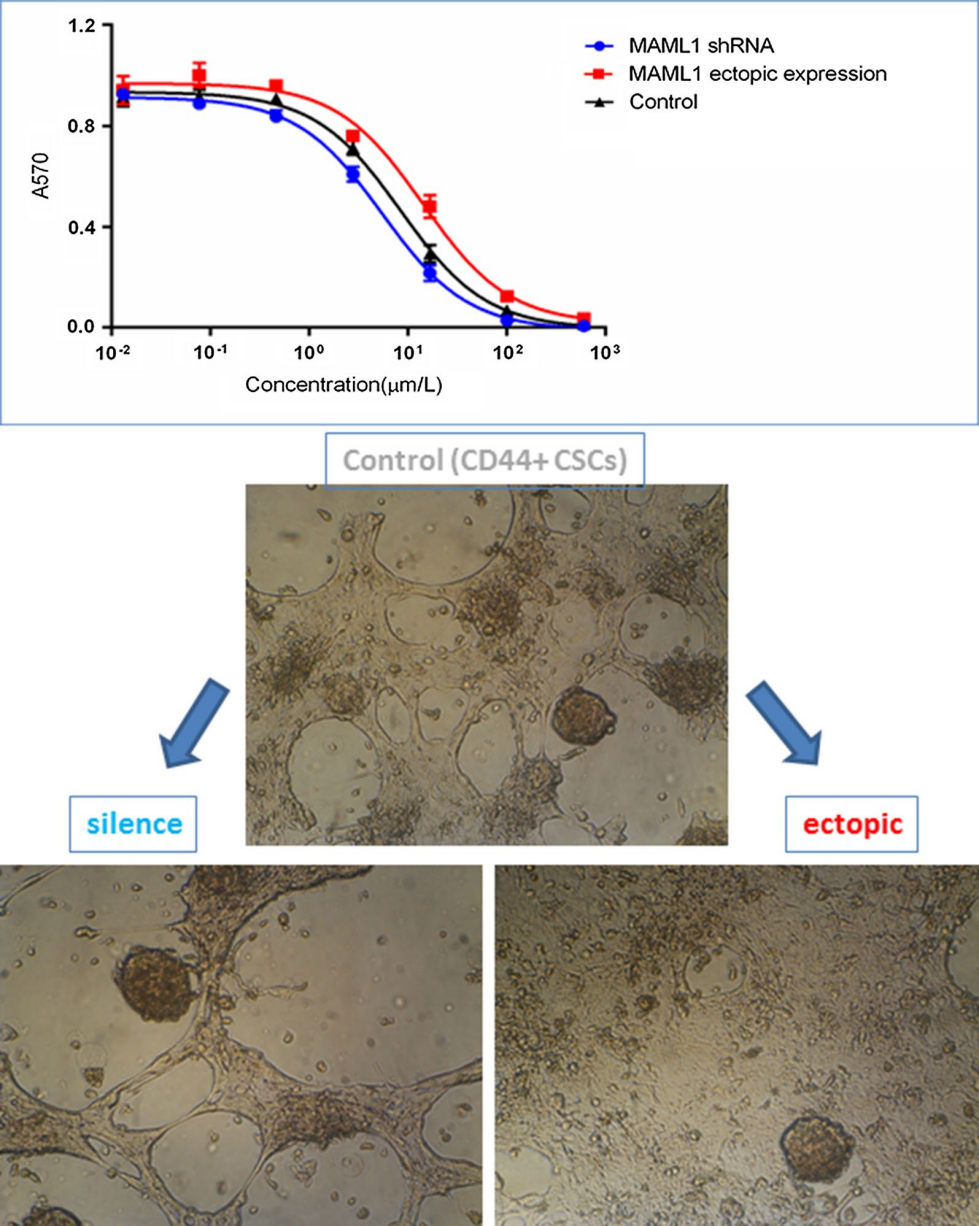
Drug resistance. Resistance toward the 5FU decreased significantly in silenced CD44+ CSCs compared with the non-transfected CD44+ CSCs. MAML1 ectopic expression also increased the 5FU resistance in CD44+ transfected cells in comparison with non-transfected CD44+ CSCs

In addition to self-renewal and EMT, resistance to chemotherapy is another feature of CSCs. Therefore, after the migration assay, we performed an MTT assay to assess the probable role of NOTCH in CD44+ CSCs drug resistance. Cancer stem cells were treated with serial dilutions of 5FU for 5 days. Resistance toward the 5FU decreased significantly in silenced CD44+ CSCs compared with the non-transfected CD44+ CSCs (5.478 µm/l vs. 8.395 µm/l, IC50) (p ≤ 0.005). MAML1 ectopic expression also increased the 5FU resistance in CD44+ transfected cells in comparison with non-transfected CD44+ CSCs (14.1 µm/l vs. 8.395 µm/l, IC50) (p ≤ 0.005) (Fig. 8). It has been shown that the ABC transporters are the main factors in drug resistance. We selected ABCG2 and ABCC4 to find the probable factors involved in 5FU resistance. MAML1 ectopic expression and silencing directly correlated with ABCG2 mRNA expression. Levels of ABCG2 mRNA in MAML1 silenced and ectopic expressed cells were − 3.72 and 2.56-fold changes respectively in comparison with the none transfected CSCs. Therefore, ABCG2 is an important factor in 5FU resistance in CD44+ CSCs, which is regulated by the NOTCH pathway in such cells.

#### Role of MAML1 in cell cycle regulation

**Fig. 9:**
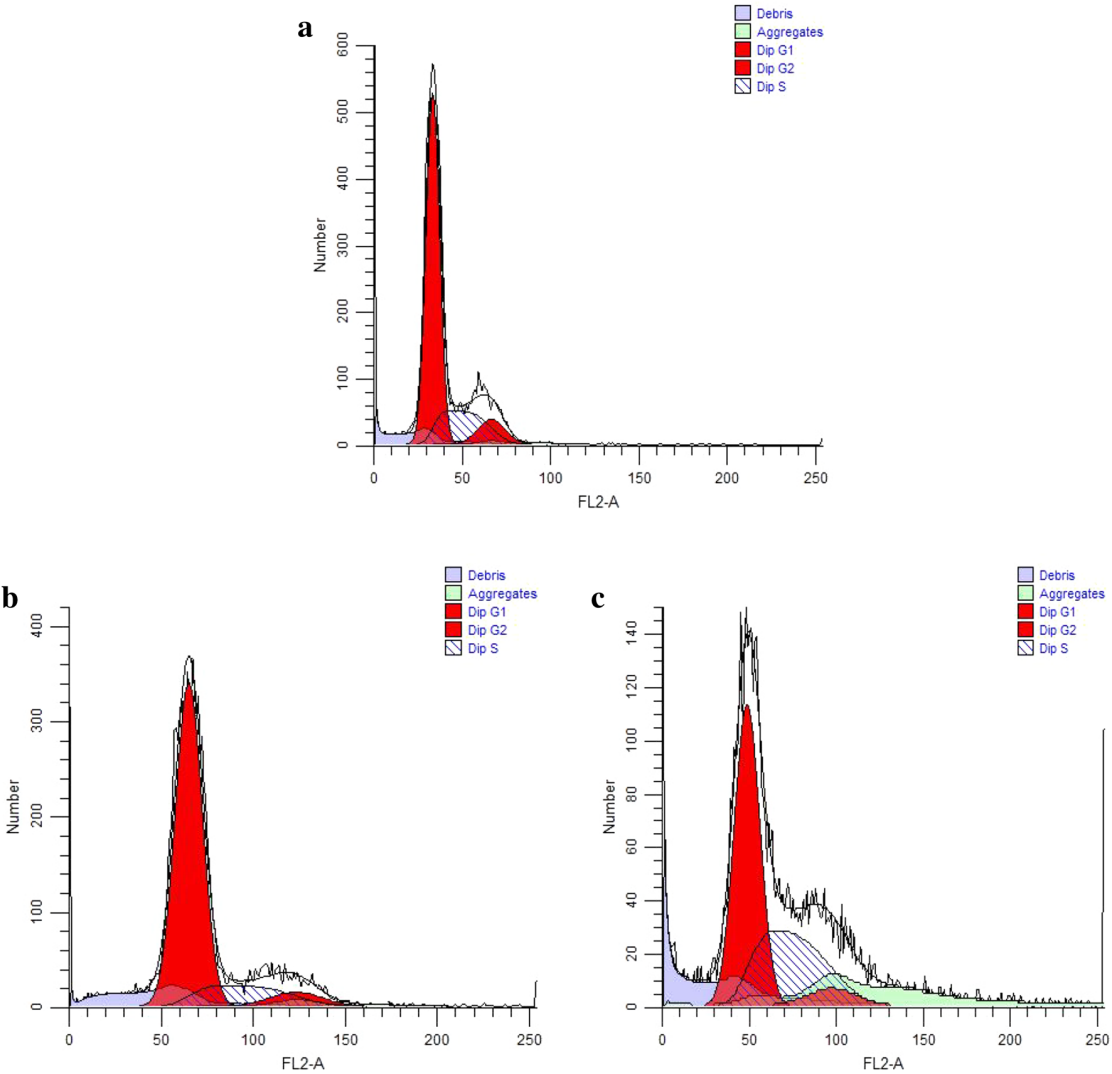
Role of MAML1 in cell cycle regulation. Cell cycle analysis using the PI flow cytometry was performed to assess and compare the percentage of transfected cells to non-transfected cells. Non transfected CSCs (**a**). MAML1 shRNA significantly increased cells in G1 phase, with a concomitant reduction in S phase (**b**). In contrast, MAML1 ectopic expression significantly increased number of cells in S phase and decreased number of cells in G1 phase (**c**)

Cell cycle analysis using the PI flowcytometry was performed to assess and compare the percentage of transfected cells to non-transfected cells. After transfection with MAML1 shRNA, CD44+ CSCs significantly accumulated at G1 phase, with a concomitant reduction in S phase (p = 0.008). Following the MAML1 silencing, number of accumulated cells in G1 phase increased 10% and number of cells in S phase decreased 6.85%. In contrast with the silencing, MAML1 ectopic expression significantly increased number of cells in S phase and decreased number of cells in G1 phase with 7.36% and 12.01%, respectively (p = 0.037) (Fig. 9). The results showed that the NOTCH signaling pathway is not only involved in drug resistance and cell migration, but also is involved in cell cycle regulation of CD44+ CSCs.

## Discussion

Clear differences in ESCC prevalence are responsible for the “Asian esophageal cancer belt”, which includes parts of China and northern Iran. Esophageal cancer in northeast of Iran is 20 times more prevalent than the other areas [30]. Cancer stem cells were first identified in leukemia, and subsequently found in solid tumors including breast, colon, prostate, liver, and melanoma [31–33]. Differentiation therapy is a well-known method to eliminate the CSCs. All-trans retinoic acid (ATRA) is one the most common differentiation therapies used to treat leukemia [34]; however, it has been shown that in addition to CSCs, ATRA stimulates normal esophageal stem cells to differentiate, leading to side effects [35, 36]. Generally, such drugs target all proliferative cells and their usage results in side effects that include weight loss, blood pressure changes, diarrhea, and heart arrhythmias. ESCC-CSCs were identified for the first time using CD44 [10]. CD44 is a universal CSC marker for various solid tumors. We previously showed a significant correlation between CD44 expression and tumor grade and depth of invasion [37]. However, CD44 expression is also observed in the basal layer of normal esophagus, which is the main location for normal esophageal stem cells [38, 39]. In this study we showed that only CD44+ CSCs are able to form tumors in NUDE mice. Moreover, these cells were more resistant to 5FU in comparison with the CD44− tumor cells. Our results indicate that the isolated CD44 cells were the CSCs. To narrow the spectrum of target cells in ESCC therapy we targeted the ESCC-CSCs via the MAML1 as the main component of the NOTCH transcription machinery. Other studies have targeted NOTCH; however, those used gamma secretase inhibitors to block ICN release inside the cytoplasm [40, 41]. Gamma secretase inhibitors also have undesirable side effects [42]; therefore, NOTCH inhibition by gamma secretase may not be an efficient method for the elimination of ESCC-CSCs. Such inhibitors target both normal and cancer stem cells. In a more precise method of targeted therapy the NOTCH receptors were targeted by specific antibodies. Targeted therapy against the NOTCH1 receptor in T-ALL resulted in fewer undesirable side effects than the gamma secretase inhibitors [43]. However, using specific antibodies also influenced the function of somatic stem cells in the basal layer [44]. Therefore, we aimed to target the CSCs with no undesirable effect on normal stem cells. Most of the recent studies targeted the NOTCH pathway at membrane receptors; however, the NOTCH pathway operates via both canonical and non-canonical processes, downstream of cell surface activation. The non-canonical pathway results in expression of MAG, a specific transcription factor for the final differentiation of normal stem cells in the basal layer [45]. The canonical pathway activates MAML1-3, which are the main components involved in expression of the HEY/HES families. MAML1, 2, and 3 maintain the stem cell features of CSCs. Therefore, canonical and non-canonical pathways are involved in biology and preservation of CSCs and normal stem cells, respectively. We previously reported that NOTCH pathway is involved in progression and metastasis of ESCC [29, 46–50]. In the present study we selected MAML1 to target the NOTCH pathway only in CSCs with the activated canonical pathway with possibly no side effects to the normal stem cells in the basal layer. CD44+ ESCC-CSCs were isolated and identified as CSCs by in vivo and molecular methods. We showed that MAML1 silencing significantly reduced cell migration. On the other hand, MAML1 ectopic expression significantly increased the metastatic behavior of isolated CSCs. Moreover, cell cycle analysis showed that MAML1 silencing in CD44+ CSCs significantly increased the number of cells in G1 phase while ectopic expression significantly increased the number of cells in S phase. We observed a correlation between MAMl1 and TWIST1 expression in isolated cells which, agreed with our recent report from studies with ESCC patients [29]. Therefore, MAML1 exerts its role in cell migration via interaction with TWIST1. Furthermore, the drug assay showed that the MAML1 ectopically-expressed cells were more resistant to 5FU than the CD44+-CSCs. MAML1 silenced cells also had lower resistance than the intact CD44+-CSCs. Moreover, beside the NOTCH pathway we tested partially the role of WNT pathway in biology of isolated cells via the PYGO2 expressional analysis. Although this marker was assessed only in the level of mRNA expression and it needs further studies, it was shown that the WNT pathway probably has not a significant role in preservation of CD44+ CSCs. WNT pathway has the major role in chemo resistance of breast CSCs via the up regulation of MDR1 expression [51]. Therefore, it seems that in contrast with breast CSCs, MDR1 and WNT pathway is not involved in chemo resistance of ESCC-CSCs and such resistance is due to the NOTCH activation and ABC transporters.

## Conclusion

We showed that the canonical NOTCH pathway in ESCC patients is one of the main factors in chemotherapeutic resistance. NOTCH pathway increases resistance toward 5FU in ESCC-CSCs using the activation of ABCG2. Therefore, targeted therapy against the canonical NOTCH pathway may be an efficient way to eliminate CSCs and decrease the rate of tumor recurrence without side effects for the normal stem cells in the basal layer.

## Abbreviations

CSCs: cancer stem cells
ESCC: esophageal squamous cell carcinoma
EC: esophageal cancer
EAC: esophageal adenocarcinoma
nCRT: neo adjuvant chemo-radiotherapy
ICN: intracellular domain of NOTCH
MTT: dimethyl thiazolyldiphenyl tetrazolium
